# Shenling Baizhu San supresses colitis associated colorectal cancer through inhibition of epithelial-mesenchymal transition and myeloid-derived suppressor infiltration

**DOI:** 10.1186/s12906-015-0649-9

**Published:** 2015-04-22

**Authors:** Xiaochang Lin, Wenjuan Xu, Meng Shao, Qin Fan, Ge Wen, Changke Li, Linlin Jing, Xuegang Sun

**Affiliations:** Department of Traditional Chinese Medicine, Nanfang Hospital, Southern Medical University, Guangzhou, 510515 China; The Key Laboratory of Molecular Biology, State Administration of Traditional Chinese Medicine; School of Traditional Chinese Medicine, Southern Medical University, Guangzhou, 510515 China; Department of Imaging Center, Nanfang hospital, Southern Medical University, Guangzhou, 510515 China; Department of Anesthesiology, Yue Bei People’s Hospital, Shaoguan, Guangdong Province China; Traditional Chinese Medicine Integrated Hospital, Southern Medical University, Guangzhou, 510315 Guangdong China

**Keywords:** Shenling Baizhu San, Colitis associated colorectal cancer, Epithelial mesenchymal transition, Myeloid-derived suppressor cells, Snail, Transforming growth factor β1

## Abstract

**Background:**

Shenling Baizhu San (SBS) is a well-known and classical Chinese medicine formula. It has been used for treatment of gastrointestinal disorders for about nine hundred years. Recent reports showed that it was effective in curing colitis and ameliorating the major manifestations of postoperational colorectal cancer (CRC). This study was to evaluate the effects of SBS on azoxymethane (AOM) and dextran sodium sulfate (DSS) induced colitis associated CRC (caCRC) and to analyze the underlying mechanism of SBS in preventing CRC.

**Methods:**

The colon tissue of mice in different group was determined by immunohistochemistry and western blot. TGF-β1 in serum was measured by ELISA. Myeloid-derived suppressor cells (MDSCs) were identified by flow cytometry and immunohistochemistry.

**Results:**

The formed neoplasms phenotypically resembled human caCRC with upregulated β-catenin, p53 and proliferating cell nuclear antigen (PCNA). SBS treatment reduced the death rate of mice and decreased the incidence and multiplicity of colonic neoplasms. SBS decreased the number of MDSCs and the level of transforming growth factor β1 (TGF-β1). SBS alleviated epithelial mesenchymal transition (EMT) through downregulating N-cadherin (N-cad), Vimentin, Fibronectin, Snail, and upregulating E-cadherin (E-cad). It reduced the activation of Wnt5a and EMT induced by TGF-β1.

**Conclusions:**

SBS reduced the death rate through decreasing the incidence and multiplicity of colonic tumors. SBS lowered MDSCs infiltration and inhibited TGF-β1 induced EMT to exert its anti-caCRC effects.

**Electronic supplementary material:**

The online version of this article (doi:10.1186/s12906-015-0649-9) contains supplementary material, which is available to authorized users.

## Background

Colorectal cancer (CRC) is the third most common cancer in men and the second in women worldwide [1]. Even after a continuous decline in the past 10 years, the estimated number of new cancer cases and deaths of CRC in the United States are 142,820 (9%) and 50,830 (9%) in 2013 [2]. Although great progress have been made in the diagnosis and treatment, surgery remains the only curative option and still 40%-50% of colorectal cancer patients died of the disease within five years of diagnosis [3]. The increasing toxicity profile and acquired chemotherapy resistance urge us to look for effective adjuvant therapies as well as improved treatment options for CRC.

Shenling Baizhu San (SBS) is a well-known and canonical Chinese medicine formula first described in “The Prescriptions of the Bureau of Taiping People’s Welfare Pharmacy” in Song-dynasty. The principle of the prescription is to replenish Qi and invigorate Spleen, resolve dampness and relieve diarrhea according to traditional Chinese medicine (TCM) theory. SBS is effective in ameliorating the major manifestations of post operational CRC, such as exhaustion and fatigue, dull and yellow complexion, anorexia, nausea, diarrhea and abdominal distension [4]. It improves Karnofsky performance scale (KPS) score and increases CD4+, CD8+ and natural killer (NK) cells in post operational CRC patients with Spleen-deficiency [5]. SBS decreases the tumor weight and increases serum interleukin 2 (IL-2), interferon-γ (IFN-γ) and tumor necrosis factor-α (TNF-α) in mice with xenografted hepatocellular cancers [6]. SBS also inhibits Lewis lung cancer growth and decreases IL-10, increases IFN-γ, NK cells and the ratio of CD4/CD8 [7]. There are evidences that SBS is effective in inhibiting or delaying the growth of CRC, hepatocellular cancer and lung cancers [5-7]. SBS is also effective in curing chronic colitis [8,9]. However, little was known if SBS can prevent colitis associated CRC (caCRC), as it is one of the three highest risk groups for developing CRC: ulcerative colitis (UC), familial adenomatous polyposis, and hereditary nonpolyposis colon cancer syndrome [10].

The epithelial-mesenchymal transition (EMT) is a process in which epithelial cells trans-differentiate and acquire an invasive mesenchymal phenotype. Activated nuclear factor-κB (NF-κB), signal transducer and activator of transcription 3 (STAT3), Ras-mitogen-activated protein kinase (MAPK), β-catenin and their controlled cytokines such as IL-1β, IL-6, IL-10, transforming growth factor-β (TGF-β) and TNF-α play pivotal roles in the formation of EMT [11]. On the other hand, the constitutive activation of MAPK, STAT3, β-catenin, and various other signaling pathways trigger the secretion of immunosuppressive molecules, e.g., TGF-β, IL-10, vascular endothelial growth factor (VEGF), and chemokine (C-C motif) ligand 2, etc. [12]. These molecules induce immunosuppressive immune cells, such as regulatory T cells (Tregs) and myeloid-derived suppressor cells (MDSCs) recruitment and accumulation in the tumor site that facilitate the development and metastasis of CRC [12]. MDSCs are a heterogeneous group of myeloid progenitor cells and immature myeloid cells that inhibit lymphocyte function by inducing regulatory T cells (Tregs); producing TGF-β and IL-10 [13]. Recently, it was reported that MDSCs suppressed antitumour immune responses and promoted cancer cell proliferation and metastasis by inducing epithelial to mesenchymal transition [14]. In our study, we found that SBS could inhibit the incidence of azoxymethane (AOM) and dextran sodium sulfate (DSS) induced caCRC. Furthermore, the underlying mechanism related to EMT and immunosuppression was also investigated.

## Methods

### SBS preparation

SBS, comprised of ten commonly used herbs: Radix Et Rhizoma Ginseng, (root of Panax ginseng C.A. Mey., Jilin, China), Poria (sclerotium of Poria cocos., Sichuan, China), Rhizoma Atractylodis Macrocephalae (root of Atractylodes macrocephala Koidz., Anhui, China), Semen Lablab Album (seed of Dolichos lablab L., Hebei, China), Rhizoma Dioscoreae (root of Dioscorea opposita Thunb., Henan, China), Radix Et Rhizoma Glycyrrhizae (root of Glycyrrhiza uralensis Fisch., Inner Mongolia, China), Plumula Nelumbinis (spire and radicle of seed of Nelumbo nucifera Gaertn., Fujian, China), Fructus Amomi (seed of Zingiberaceae, Amomum villosum Lour., Yangchun, China), Semen Coicis (nut of Coix lachrhryma-jobi L.var.mayuen (Roman) Stapf., Fujian, China), Radix Platycodonis (root of Platycodon grandiflorum(Jacq.)A.DC., Anhui, China). The raw herbs for SBS were purchased from the affiliated Nan Fang Hospital of Southern Medical University. The herbs were identified as genuine regional drug by Prof Chen Xingxing, an expert of Chinese medicine identification, Southern Medical University. A voucher specimen was deposited in the Herbarium of school of traditional Chinese medicine. These were mixed in the ratio of 15:15:15:12:15:9:9:6:9:6 (dry weight). Aqueous extracts of SBS were extracted at 80°C by stirring it for 1 h using 10 volumes of distilled water (v/m). Then, we centrifuged the extract at 1,500 × g at room temperature. To obtain the semisolid SBS solution, the supernatant was collected and subjected to condensation under reduced pressure at 70°C. The quality of SBS was controlled by HPLC analysis (Additional file 1: Figure S1, Table S1, Figure S2). SBS was suspended again in 0.9% saline at a final concentration of 2 g/mL. The solution was stored in aliquots at −20°C.

### Animals and experimental procedure

All procedures involving laboratory animals were conducted in accordance with the guidelines of the Instituted Animal Care and Use Committee of Southern Medical University. All protocols were submitted and validated by Animal Care Ethics Committee of Southern Medical University (No. 2012–055). Male C57BL/6 J mice (specific pathogen-free) were obtained from the Laboratory Animal Center of Southern Medical University. The animals were maintained under controlled conditions (22°C, 12-h/12-h dark/light cycle) in a conventional animal colony. The mice were between 6 and 8 weeks old at the beginning of the experiments, and weighed 15–20 g. There were 65 mice used in the study, 15 mice in normal control (control) group, 30 in AOM/DSS (model) group and 20 in AOM/DSS & SBS (SBS) group. At the end of the study, there were 15 surviving mice in control group, 9 in model group and 12 in SBS group, respectively.

**Figure 1:**
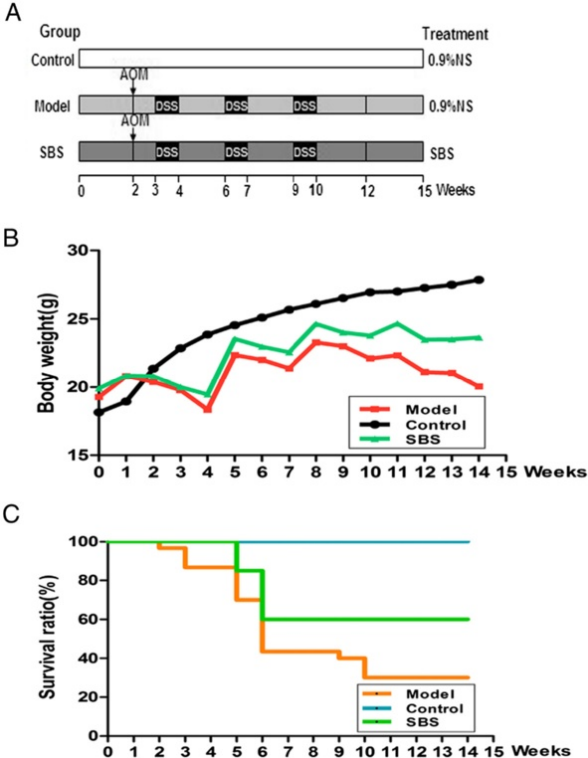
Effects of SBS on the general well being of mice treated with AOM/DSS. **(A)** Experimental protocol for colitis-associated colon carcinogenesis model. **(B)** Effect of SBS on body weight of mice. **(C)** Effect of SBS on the survival ratio of mice. SBS enhanced animal survival ratio (*P* = 0.04, vs. Model). Data are presented as mean ± SD vs. control and model.

Procedures of induction of caCRC model by AOM and DSS were shown in Figure 1A [15,16]. At week 2, C57BL/6 J mice were injected with AOM (10 mg · kg^−1^, i.p.). 1 week later, 3% DSS (International Lab, Chicago, IL, USA) was added to the drinking water for 7 days followed by 14 days of tap water for recovery. This cycle was repeated twice.

SBS (7.28 g/kg) or equivalent normal saline was administered by gavage using a tube twice a day. The mice were killed by cervical dislocation at week 16 and the colons (from the ileocecal junction to the anal verge) were removed. After measuring the length and weight, the colons were cut open longitudinally along the main axis, washed with phosphate-buffered saline (pH 7.4, 4°C) and then macroscopically inspected. The number, size and location of pre-neoplastic and neoplastic lesions (dysplasia and carcinoma) in the colons were recorded based on gross examination. The sizes of dysplasia were measured by an ocular micrometer. After gross examination, the colons were cut into pieces at about 1 cm intervals. One third of colon samples were fixed in 10% buffered formalin (pH 7.4); one third was kept in 10 volumes of RNAlater Solution (Ambion, Life technologies, Carlsbad, CA, USA) and the remaining one third was flash-frozen in liquid nitrogen and stored at −80°C for further immunohistochemistry, PCR and protein analysis, respectively.

### Reagents

Ginsenoside Rc (GRc) and Atractylenolide-1 (AT1) were purchased from CHENGDU MUST BIO-TECHNOLOGY Co., Ltd. Dimethyl sulfoxide (DMSO) and thiazolyl blue tetrazolium bromide (MTT) were purchased from Sigma-Aldrich (St. Louis, MO, USA). RPMI 1640, FBS and antibiotics were purchased from Invitrogen (Gibco, Grand Island,NY, USA). Recombinant Human TGF-β1 was purchased from Whiga (PeproTech Inc, USA).

### Cell viability assays

MTT assay was used to analyze the effects of GRc , AT1 and SBS on cell viability in two kinds of colorectal adenocarcinoma cell lines SW480,HCT116. Cells were routinely maintained in RPMI 1640, supplemented with 10% FBS and antibiotics (50 U/ml of penicillin and 50 μg/ml streptomycin) at 37°C in a humidified atmosphere containing 5% CO2. Cell number was measured on a standard microscope by manual counting of cells using a grid with an area of 0.25 mm^2^. 1 × 10^4^ Cells were seeded on 96-well plates in a 100 μl volume. The MTT assay was performed by addition of MTT to a final concentration of 0.5 mg/ml of medium. After 4 h, the medium was removed, and 150 μl of DMSO was added to each well. After 10 min of shaking at room temperature, 150 μl of soluble material was placed into a new 96-well plate, and absorbance at 562 nm was read with a background subtraction at 660 nm.

### Preparation of MDSCs

Spleen and tumors were collected and cut into small pieces before incubation for 15 min at 37°C with the following enzymes dissolved in HBSS: collagenase Type I (0.05 mg/ml), collagenase Type IV (0.05 mg/ml), hyaluronidase (0.025 mg/ml), and DNase I (0.01 mg/ml) and soybean trypsin inhibitor (0.2 trypsin inhibitor unit/ml). The digested cells were harvested, and the RBCs were lysed by using hypotonic buffer (0.155 M NH4Cl, 0.1 mM EDTA, 10 mM KHCO3) for 1 min. CD11b^+^ cells were isolated by using anti-CD11b magnetic immunobeads according to the manufacturer’s instructions (MACS, Miltenyi Biotec, Berdish-Gladbach, Germany).

### Cytokine measurement by enzyme-linked immunosorbent assay

The level of TGF-β1 in serum was measured using mice TGF-β1 Elisa kit (Novatein Biosciences, Woburn, MA, USA) according to its protocol.

### Flow cytometric analysis of MDSCs

To analyze CD11b^+^ cell surface antigen expression, allophycocyanin (APC)-conjugated anti-CD11b (M1/70, PharMingen) and PE-conjugated anti-Ly6g (Gr-1; RB6-8C5, PharMingen) were used. All antibodies were used at 10 μg/ml. The cells were incubated with the antibodies for 30 min at 4°C and washed with PBS. The samples were fixed with 1% paraformaldehyde/PBS and analyzed by using a FACSCalibur flow cytometer and CellQuest software (Becton Dickinson Japan, Tokyo).

### Histopathological assessment

For histopathological examination, formalin fixed, paraffin-embedded colon tissues were cut into serial sections (5 μm) and stained with haematoxylin and eosin (H&E) staining. Histological alterations such as dysplasia and carcinoma were verified. Assessment of dysplasia and adenocarcinoma was based on the criteria set forth in the Mouse Models of Intestinal Cancers consensus report: location, presence/absence of prolapse/herniation, size (in mm), dysplasia grade, invasion level, presence of desmoplasia, and presence of irregular architecture [10]. Carcinoma was defined as high-grade dysplasia of colonic mucosa which invaded beyond the muscularis mucosa and into the submucosa [15].

### Immunohistochemistry

Paraffin-embedded colon sections were dewaxed, rehydrated, and pre-treated with hydrogen peroxidase in PBS buffer. Heat-induced antigen retrieval was performed. After blocking with the appropriate antisera in blocking buffer, sections were incubated with anti-Proliferating cell nuclear antigen (PCNA) (Thermo scientific, clone PC10, 1:300 dilution), anti-β-catenin (Cell Signaling Technology, CST, clone 6B3, 1:100 dilution), anti-p53 (Leica-microsystems, clone CM5, 1:100 dilution), or anti-COX-2 (Thermo scientific, 1:100 dilution), anti-E-cadherin (CST, clone 24E10, 1:200 dilution), anti-N-cadherin (Millipore, clone EPR1792Y, 1:50 dilution), anti-Fibronectin (Epitomics, clone F14, 1:200 dilution) and anti-Vimentin (CST, clone D21H3, 1:50 dilution). After incubation with HRP-conjugated secondary antibody and tyramide amplification followed by streptavidin-HRP, positive signals were visualized by DAB kit , and counter-stained with hematoxylin. Double staining of CD11b^+^Ly6g^+^ cells was carried out using Polink RRt DouSP kit (ZSGB-BIO ORIGENE, China). CD11b (abcam, clone EPR1344, 1:100 dilution), Ly6g (abcam, clone RB6-8C5, 1:20 dilution). Five randomly selected fields from each section were examined at a magnification of 400× and analyzed using NIS-Elements. The positive content was calculated using the following formula: positive content (PC) = mean optical density × positive area.

### Western blot analysis

Aliquots of Colonic tissues were homogenized in liquid nitrogen, dissolved in lysis buffer [7 mol/L urea, 2 mol/L thiourea, 4% (w/v) CHAPS, 20 mmol/L Tris, 65 mmol/L DTT, 0.2% pharmalyte 3/10 ampholyte] supplemented with 8 μl of protease inhibitor cocktail (The protease inhibitor cocktail include AEBSF hydrochloride 500 μM, Aprotinin 150nM, E-64 protease inhibitor 1 μM, EDTA disodium 0.5 mM and Leupetin hemisulfate 1 μM. Calbiochem, San Diego, CA, USA). The samples were then centrifuged at 15,000 × g for 30 min at 4°C. Protein concentrations were determined with modified Bradford assay. The protein lysates were loaded onto 10% SDS-PAGE for separation and electrotransferred to PVDF membranes and blocked in 5% non-fat milk in Tris-buffered saline (TBST, 100 mM NaCl, 50 mM Tris, 0.1% Tween-20, pH 7.5). Membranes were incubated overnight with primary antibodies [anti-PCNA (CST, clone PC10, 1:1000 dilution), anti-β-catenin (CST, clone 6B3, 1:1000 dilution), anti-p53 (Leica-microsystems, clone CM5, 1:1000 dilution), or anti-COX-2 (CST, clone D5H5 1:1000 dilution), anti-E-cadherin (CST, clone 24E10; 1:1000 dilution), anti-N-cadherin (Millipore, clone EPR1792Y, 1:5000 dilution), anti-Vimentin (CST, clone D21H3, 1:1000 dilution), anti-Snail (CST, clone C15D3, 1:1000 dilution), anti-Frizzled (Santa Cruz, clone F-13, 1:1000 dilution), anti-Dishevelled-2 (CST, clone 30D2; 1:1000 dilution), anti-Glycogen synthase kinase 3β (GSK-3β) (CST, clone 27C10, 1:1000 dilution), anti-phosphorylated GSK-3β (Serine 9) (CST, clone Ser9, 1:1000 dilution), anti-Wnt5a (CST, clone C27E8, 1:1000 dilution)] at 4°C. This was followed by incubation with horseradish peroxidase (HRP) conjugated secondary antibodies. The results were visualized with chemiluminescence (ECL, GE Healthcare Bio-science, Uppsata, Sweden). The images were captured and documented with a CCD system (imagestation 2000MM, Kodak, Rochester, NY, USA). The quantitative analysis of these images was performed using Molecular Imaging Software Version 4.0, provided by Kodak 2000 MM System. The optical density was normalized against that of β-actin [16].

### Statistical analysis

Each experiment was repeated at least three times. Data were presented as mean ± SD. All data were analyzed using the SPSS statistical package (version 13.0, SPSS Inc, Chicago, IL, USA). Survival rate was analyzed with Kaplan-Meier survival analysis. Data between two groups were compared with 2-independent samples tests. Mean values of data from more than 3 groups were compared with one-way analysis of variance (ANOVA) and multi-comparison was performed. The correlation analysis was determined by Spearman’s correlation coefficient. A value of *P* < 0.05 was considered as statistically significant.

## Results

### Effects of SBS on the general well being of mice treated with AOM/DSS

AOM/DSS was used to induce caCRC and effects of SBS on caCRC were evaluated (Figure 1A). Body weight loss and bloody stools were observed in mice exposed to DSS (Figure 1B). These symptoms were relieved during the recovery periods. The mean colon weights were increased and the mean colon length were decreased significantly in mice receiving AOM/DSS (Table 1) as compared to the control group. The significant increase in colon weight to colon length ratio was a result of apparent mucosal thickening.

**Table 1:** **Colon and spleen assessment in mice**

	**Colon**			**Spleen**	
**Group**	**Weight (g)**	**Length (cm)**	**W/L ratio**	**Weight (g)**	**SW/BW ratio**
Model (n = 9)	0.52 ± 0.094^△^	5.70 ± 0.444^△^	0.09 ± 0.018^△^	0.35 ± 0.039^△^	0.018 ± 0.0021^△^
Control (n = 15)	0.23 ± 0.020	9.08 ± 0.521	0.02 ± 0.002	0.07 ± 0.006	0.003 ± 0.0002
SBS (n = 12)	0.35 ± 0.064^*^	7.77 ± 0.548^*^	0.05 ± 0.009^*^	0.17 ± 0.031^*^	0.007 ± 0.0015^*^

*P < 0.01 vs. model.

^△^P < 0.01 vs. control.

A dose gradient of SBS (3.64 g/kg, 7.28 g/kg, 14.56 g/kg) was pre-screened. The medium dose SBS (7.28 g/kg) was the selected optimal dose with satisfactory efficacy for it retarded the development of neoplasms significantly (Additional file 2). The body weight fluctuation and mucosal thickening were substantially alleviated in mice receiving SBS treatment. Based on Kaplan-Meier survival curves (Figure 1C), SBS treatment significantly increased the survival rate of mice.

### SBS reduced the incidence and multiplicity of colonic neoplasms

**Figure 2:**
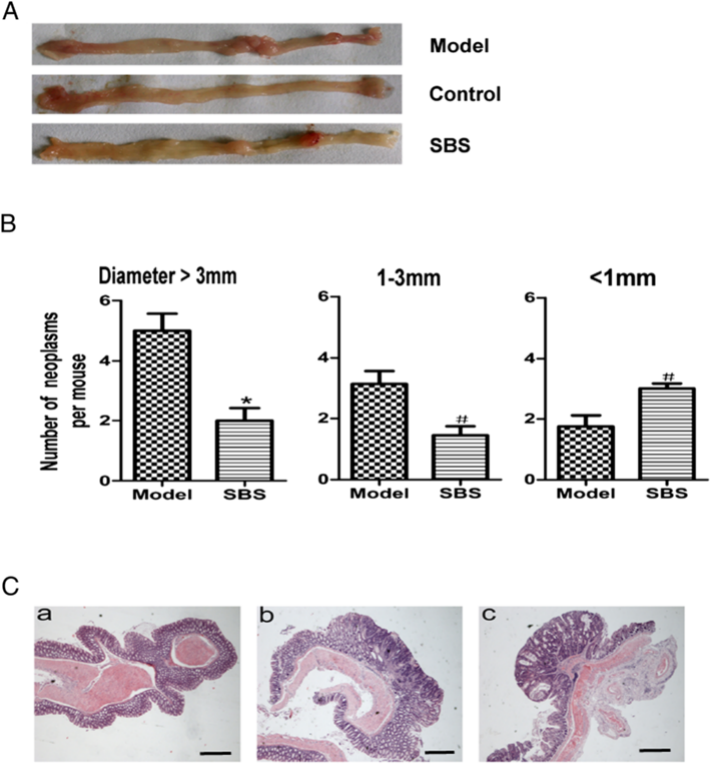
SBS reduced the incidence and multiplicity of colonic neoplasms. **(A)** Microscopic view of colon in mice. **(B)** Effect of SBS on colonic neoplasms size. SBS significantly delayed tumor growth and reduced neoplasms size. Data were presented as mean ± SD. ^*^ *P* < 0.01 vs. model, ^#^ *P* < 0.05 vs. model. **(C)** Most of colorectal neoplasms were histologically consistent with tubular adenoma or adenocarcinoma. Histological studies were carried out based on haematoxylin and eosin (H&E) staining as described. (Ca) Normal colon; (Cb) tubular adenoma, (Cc) adenocarcinoma. 40× for all, scale bar = 100 μm.

AOM/DSS treatment resulted in 100% incidence of colonic neoplasms. Most of the neoplasms were distributed in the middle and distal colon (Figure 2A). SBS treatment decreased the total multiplicity of colorectal neoplasms by 34.8% (*P* < 0.01). It is noteworthy that SBS significantly retarded the development of large neoplasms (diameter >3 mm) by 60% (Figure 2B). Histological assessment showed that most of the lesions in the colon were tubular adenoma or adenocarcinoma according to criteria of Mouse Models of Intestinal Cancers (Figure 2C).

**Figure 3:**
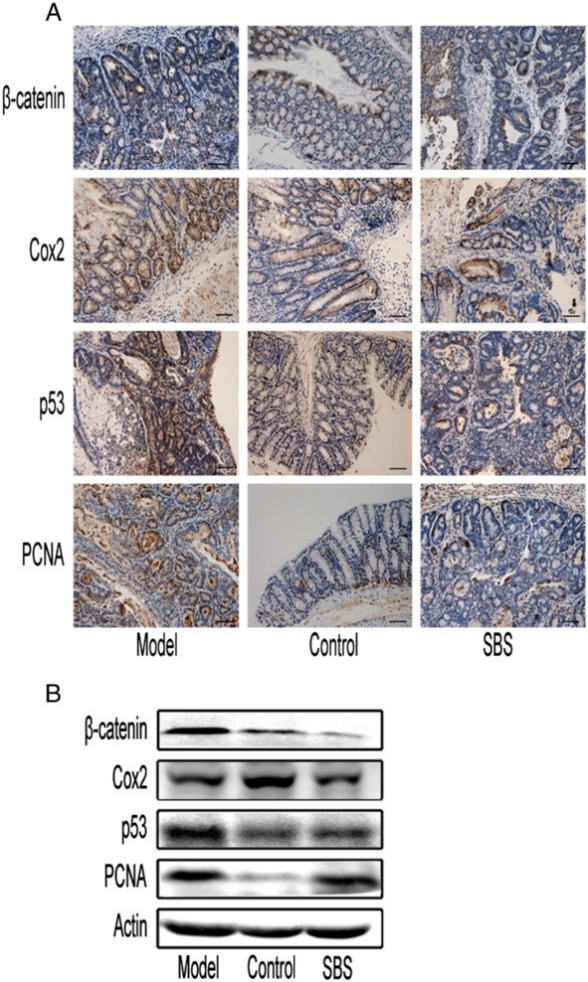
Effects of SBS on oncogenic protein expression. **(A)** Immunohistochemical staining of β-catenin, COX-2, p53 and PCNA expression in tumor tissues or normal colonic tissues. 200× for all, scale bar = 100 μm. SBS significantly decreased the expression of β-catenin, p53 and PCNA, respectively, but not COX-2. **(B)** Western blot analysis of β-catenin, COX-2, p53, PCNA expression in colonic tissues. The expression of these proteins was reduced in SBS group compared with model group, except COX-2. Semiquantitive analysis of these proteins is showed in Tables 2 and 3.

To determine whether AOM/DSS induced caCRC phenotypically resembles human caCRC, we assessed the expression of neoplastic markers by immunohistochemistry. Similar to human caCRC, PCNA and β-catenin expression were increased in dysplastic and neoplastic lesions (Figure 3A-B, Tables 2 and 3). Intense nuclear staining for p53 (CM5 clone: detects both mutant and wild-type forms) was detected in the neoplastic epithelium and in nondysplastic crypts; an observation that is highly suggestive of p53 mutations [10]. However, no high-level expression of COX-2 in adenocarcinoma was observed (Figure 3A-B, Tables 2 and 3).

**Table 2:** **Semiquantitive analysis of Immunohistochemical staining**

	**Control (n = 15)**	**Model (n = 9)**	**SBS (n = 12)**
β-catenin	0.07 ± 0.06	5.74 ± 2.87^▲^	1.07 ± 0.91^#^
COX-2	1.23 ± 0.89	1.87 ± 1.25	2.07 ± 1.46
p53	0.97 ± 1.24	6.71 ± 3.81^△^	2.67 ± 2.58^#^
PCNA	0.97 ± 1.13	6.00 ± 3.01^△^	2.91 ± 1.84^#^
E-cadherin	3.37 ± 1.82	0.06 ± 0.04^▲^	4.76 ± 2.18^#^
N-cadherin	0.92 ± 0.75	7.09 ± 2.90^△^	3.34 ± 2.47^#^
Fibronectin	1.06 ± 1.08	9.07 ± 3.87^△^	4.88 ± 3.10^#^
Vimentin	1.03 ± 1.07	6.49 ± 3.32^△^	3.10 ± 2.35^#^
CD11b^+^Ly6g^+^	0.03 ± 0.04	2.82 ± 0.95^△^	1.00 ± 0.64^#^

^#^P < 0.05 vs. model.

^△^P < 0.01 vs. control; ^▲^P < 0.05 vs. control.

**Table 3:** **Semiquantitive analysis of Western blot**

	**Control (n = 15)**	**Model (n = 9)**	**SBS (n = 12)**
β-catenin	0.11 ± 0.03	0.36 ± 0.08^△^	0.08 ± 0.02^*^
COX-2	0.52 ± 0.05	0.31 ± 0.07^△^	0.27 ± 0.03
p53	0.15 ± 0.06	0.69 ± 0.08^△^	0.36 ± 0.06^*^
PCNA	0.09 ± 0.01	0.57 ± 0.04^△^	0.32 ± 0.04^*^
E-cadherin	0.81 ± 0.06	0.28 ± 0.03^△^	0.45 ± 0.09^#^
N-cadherin	0.16 ± 0.05	0.71 ± 0.12^△^	0.48 ± 0.07^#^
Snail	0.13 ± 0.05	0.56 ± 0.11^△^	0.38 ± 0.09^#^
Vimentin	0.15 ± 0.06	0.73 ± 0.04^△^	0.42 ± 0.26^*^
Frizzled	0.08 ± 0.02	0.17 ± 0.07^▲^	0.07 ± 0.02^#^
Axin	0.52 ± 0.09	0.08 ± 0.04^△^	0.32 ± 0.05^*^
Dvl2	0.66 ± 0.05	0.17 ± 0.04^△^	0.44 ± 0.10^*^
P-Gsk-3β	0.32 ± 0.05	0.15 ± 0.08^▲^	0.55 ± 0.07^*^
Gsk-3β	0.34 ± 0.06	0.36 ± 0.11	0.35 ± 0.11
Wnt5a	0.18 ± 0.03	0.66 ± 0.06^△^	0.42 ± 0.05^*^

*P < 0.01 vs. model; ^#^P < 0.05 vs. model.

^△^P < 0.01 vs. control; ^▲^P < 0.05 vs. control.

### SBS and AT1 reduced CRC cell viability

**Figure 4:**
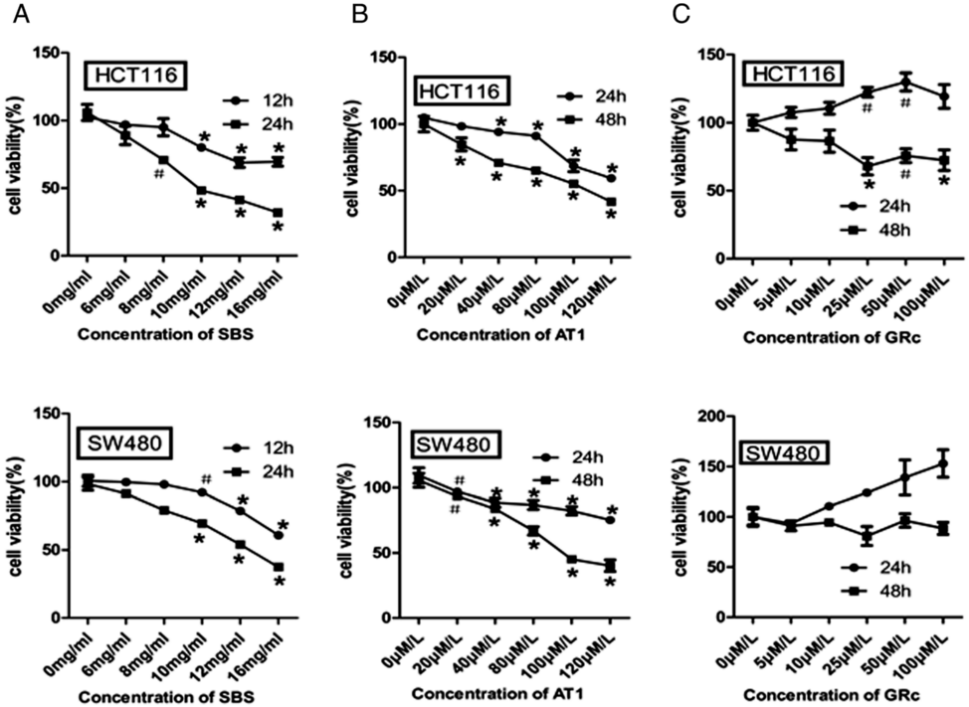
Effects of SBS, AT1 and GRc on colorectal cancer cells in vitro. **(A)** Effects of SBS on proliferation of SW480 and HCT116. Cells were exposed to 0–16 mg/mL SBS and incubated for 12 and 24 h. Cell viability was measured by MTT methods and values were expressed as percentage of the cell viability compared to 0-only. *P < 0.01 vs. 0 mg/mL; #P < 0.05 vs. 0 mg/mL. **(B)** Effects of AT1 on proliferation of SW480 and HCT116. Cells were exposed to 0–120 μM/L AT1 and incubated for 24 and 48 h. Cell viability was measured by MTT methods and values were expressed as percentage of the cell viability compared to 0-only. *P < 0.01 vs. 0 μM/L; #P < 0.05 vs. 0 μM/L. **(C)** Effects of GRc on proliferation of SW480 and HCT116. Cells were exposed to 0–100 μM/L GRc and incubated for 24 and 48 h. Cell viability was measured by MTT methods and values were expressed as percentage of the cell viability compared to 0-only. *P < 0.01 vs. 0 μM/L; #P < 0.05 vs. 0 μM/L.

To further analyze the anti-tumor effects of SBS, cell proliferation was evaluated in SW480 and HCT116 cells treated with SBS and its two ingredients, AT1 and GRc. SBS significantly decreased cell viability in a time- and dose-dependent manner from 10 mg/mL to 16 mg/mL. The viabilities of SW480 and HCT116 cells decreased to 53.8714% and 41.2625% after 24 h of treatment with 12 mg/mL SBS (Figure 4A). AT1, but not GRc, significantly decreased cell viability in a time-and dose-dependent manner. The viabilities of SW480 and HCT116 cells decreased to 44.9733% and 55.032% after 48 h of treatment with 100 μM/L AT1 (Figure 4B).

### SBS decreased MDSCs infiltration and reduced TGF-β1

**Figure 5:**
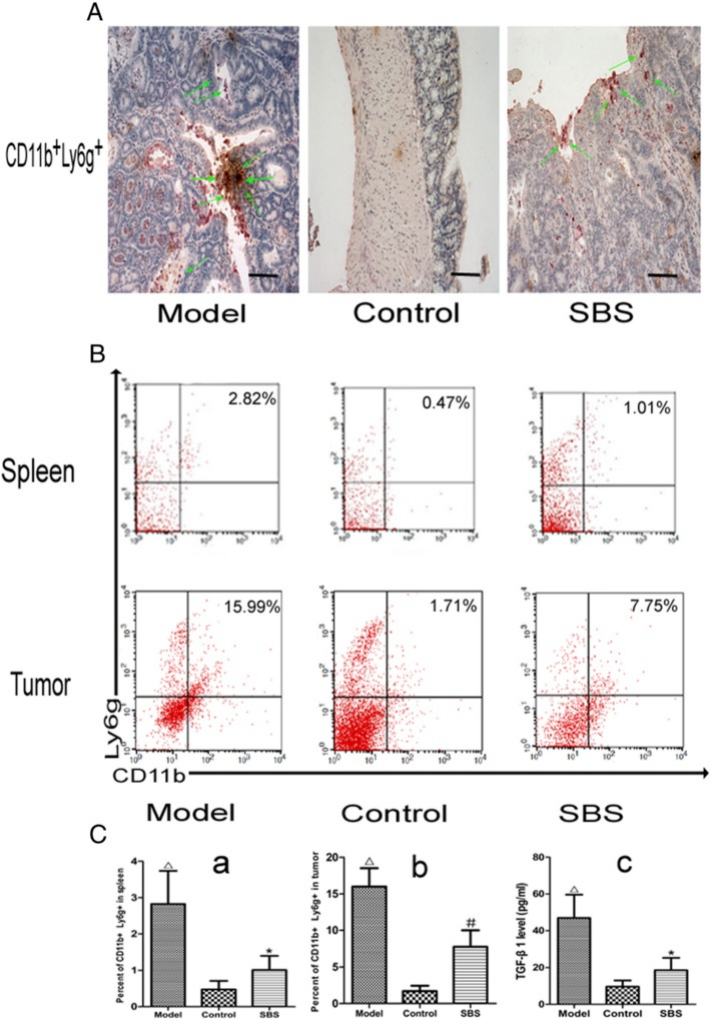
Effects of SBS on MDSCs and serum TGF-β1. **(A)** Immunohistochemical double staining of MDSCs in tumor tissues or normal colonic tissues. 200 × for all, scale bar = 100 μm. SBS decreased the expression of MDSCs. Semiquantitive analysis is showed in Table 2. **(B)** Flow cytometry analysis of MDSCs in spleen and tumor. SBS decreased the number of MDSCs versus model group. **(C)** Enzyme-linked immunosorbent assay of serum TGF-β1 and semiquantitive analysis of Flow cytometry analysis of MDSCs. Serum contents of TGF-β1 was reduced by SBS. Data are presented as mean ± SD. ^*^ *P* < 0.01 vs. model, ^#^ *P* < 0.05 vs. model; ^△^ *P* < 0.01 vs. control, ^▲^ *P* < 0.0 5 vs. control.

Immunohistochemical double staining of CD11b^+^Ly6g^+^ cell showed that the number of MDSCs was increased in model group and decreased by SBS treatment (Figure 5A, Table 2). This result was corfirmed by Flow cytometric analysis of CD11b^+^Ly6g^+^ cell in spleen and tumor (Figure 5B-C_a-b_). TGF-β1, the major effector molecule of MDSCs, was increased in model group and decreased by SBS treatment (Figure 5Cc).

### The effects of SBS on expression of EMT markers and Wnt pathway molecules

**Figure 6:**
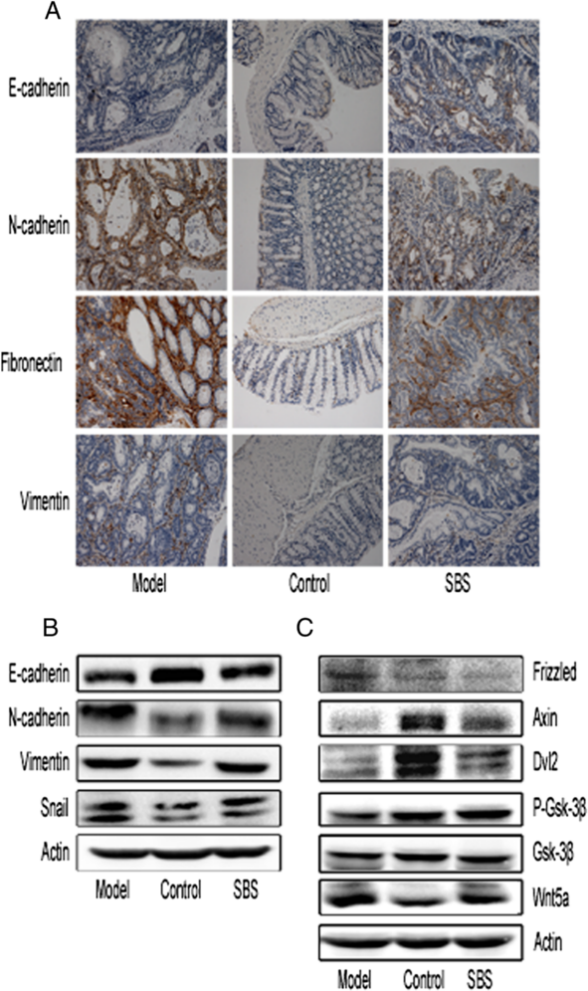
Effects of SBS on EMT markers and Wnt pathway molecules. **(A)** Immunohistochemical staining of E-cadherin, N-cadherin, Fibronectin, Vimentin in tumor tissues or normal colonic tissues. 200 × for all, scale bar = 100 μm. SBS increased the E-cad expression, decreased N-cadherin, Fibronectin, Vimentin expression. **(B)** Western blot of E-cadherin, N-cadherin, Vimentin, Snail in colonic tissues. E-cadherin was significantly deficient in model group. The expression of E-cadherin was up-regulated and the expression of N-cadherin, Fibronectin, Vimentin were downregulated by SBS treatment. **(C)** Western blot analysis of Wnt pathway molecules. SBS increased the expression of Dvl2, Axin, and p-GSK-3β, decreased Wnt5a. Semiquantitive analysis of EMT markers is showed in Tables 2 and 3.

TGF-β1 induces epithelial mesenchymal transition and increases the stablization of β-catenin [17], the effects of SBS on EMT and Wnt pathway were tested. A constitutive expression of E-cadherin was found in epithelials of the control group. In accordance with the occurrence of EMT, E-cadherin expression was downregulated in model group. SBS increased the E-cad expression moderately (Figure 6A, Table 2). Furthermore, the other three EMT markers, N-cad, Fibronectin and Vimentin were strongly expressed in model group and were decreased by SBS significantly (Figure 6A, Table 2). Western blot analysis confirmed increased expression of E-cad, and decrease of N-cad and Vimentin. We also found that upregulation of Snail, a transcription factor that plays a pivotal role in the formation of EMT, was decreased by SBS (Figure 6B, Table 3).

Wnt5a was increased in model group and was decreased by SBS while Wnt3a was not detected. SBS increased the expression of Axin, Disheveled 2 (Dvl2). The phosphorylation of glycogen synthase kinase-3β (GSK-3β) at serine 9 was elevated in control group and reduced in model group, SBS increased the phosphorylation of GSK-3β moderately (Figure 6C, Table 3).

**Figure 7:**
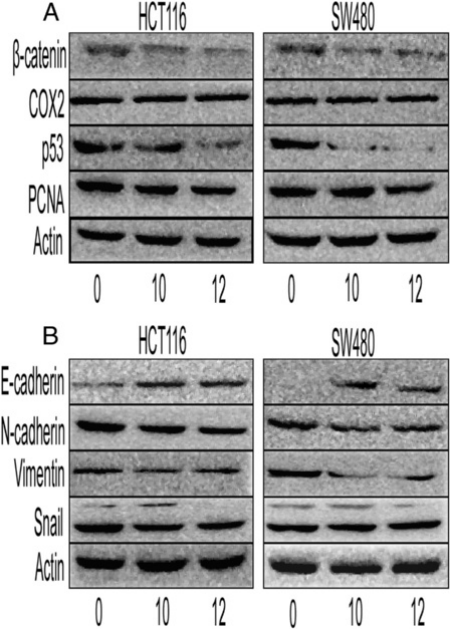
Effects of SBS of oncogenic protein and EMT marker expression on colorectal cancer cells, HCT116, SW480. **(A)** Western blot of β-catenin, COX-2, p53 and PCNA expression on SW480 and HCT116 exposed to 0, 10, 12 mg/mL SBS. The expression levels of these proteins were reduced in a dose-dependent manner. Semiquantitive analysis of these proteins is showed in Tables 4 and 5. **(B)** Western blot of E-cad, N-cad, Vimentin and Snail expression on SW480 and HCT116 exposed to 0, 10, 12 mg/mL SBS. The expression levels of N-cad, Vimentin and Snail were reduced in a dose-dependent manner, while E-cad was increased in a dose-dependent manner. Semiquantitive analysis of these proteins is showed in Tables 4 and 5.

**Figure 8:**
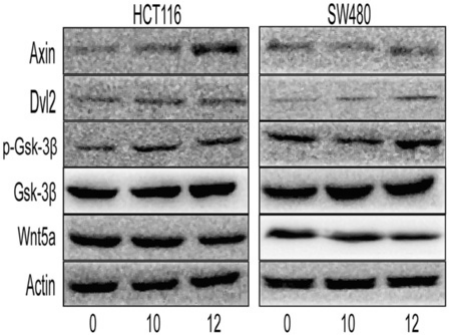
Effects of SBS of Wnt pathway molecules on colorectal cancer cells, HCT116 and SW480. **(A)** Western blot of Axin, Dvl2, p-Gsk-3β and Gsk-3β expression on SW480 and HCT116 exposed to 0, 10, 12 mg/mL SBS. Semiquantitive analysis of these proteins is showed in Tables 4 and 5.

The *in vivo* results were further confirmed in CRC cell lines. SBS decreased the expression levels of PCNA, β-catenin, p53 in a dose-dependent manner (Figure 7A, Tables 4 and 5). SBS increased the E-cad expression significantly, and decreased the expression of N-cad, Vimentin and Snail in a dose-dependent manner (Figure 7B, Tables 4 and 5). It increased the expression of Axin, Dvl2 and glycogen synthase kinase-3β (GSK-3β) in a dose-dependent manner. The phosphorylation of glycogen synthase kinase-3β (GSK-3β) at serine 9 was increased in SBS group (Figure 8, Tables 4 and 5). Furthermore, the Spearman’s correlation analysis showed that β-catenin negatively correlated with p-GSK-3β (r = −0.818, P = 0.007).

**Table 4:** **Semiquantitive analysis of Western blot on HCT116**

	**0 (n = 3)**	**10 (n = 3)**	**12 (n = 3)**
β-catenin	0.34 ± 0.03	0.15 ± 0.02^*^	0.03 ± 0.02^*^
COX-2	0.57 ± 0.05	0.53 ± 0.04	0.55 ± 0.03
p53	0.74 ± 0.06	0.48 ± 0.08^*^	0.25 ± 0.04^*^
PCNA	0.87 ± 0.04	0.66 ± 0.04^*^	0.61 ± 0.04^*^
E-cadherin	0.08 ± 0.01	0.33 ± 0.02^*^	0.34 ± 0.05^*^
N-cadherin	0.84 ± 0.06	0.62 ± 0.03^*^	0.52 ± 0.03^*^
Snail	0.84 ± 0.04	0.74 ± 0.02^*^	0.63 ± 0.05^*^
Vimentin	0.43 ± 0.05	0.31 ± 0.02^#^	0.31 ± 0.05^#^
Axin	0.01 ± 0.01	0.17 ± 0.04^*^	0.33 ± 0.04^*^
Dvl2	0.10 ± 0.04	0.14 ± 0.04	0.22 ± 0.04^*^
p-Gsk-3β	0.22 ± 0.05	0.42 ± 0.05^*^	0.52 ± 0.04^*^
Gsk-3β	0.74 ± 0.04	0.84 ± 0.03^#^	0.89 ± 0.04^*^

*P < 0.01 vs. 0 mg/mL; ^#^P < 0.05 vs.0 mg/mL.

**Table 5:** **Semiquantitive analysis of Western blot on SW480**

	**0 (n = 3)**	**10 (n = 3)**	**12 (n = 3)**
β-catenin	0.38 ± 0.05	0.23 ± 0.04^*^	0.23 ± 0.03^*^
COX-2	0.50 ± 0.03	0.49 ± 0.07	0.55 ± 0.03
p53	0.70 ± 0.03	0.17 ± 0.04^*^	0.14 ± 0.04^*^
PCNA	0.83 ± 0.05	0.71 ± 0.07^#^	0.59 ± 0.04^*^
E-cadherin	0.01 ± 0.01	0.23 ± 0.04^*^	0.27 ± 0.06^*^
N-cadherin	0.53 ± 0.05	0.36 ± 0.04^*^	0.32 ± 0.06^*^
Snail	0.63 ± 0.05	0.42 ± 0.04^*^	0.42 ± 0.06^*^
Vimentin	0.58 ± 0.05	0.29 ± 0.08^*^	0.28 ± 0.03^*^
Axin	0.08 ± 0.01	0.09 ± 0.03	0.23 ± 0.05^*^
Dvl2	0.09 ± 0.04	0.11 ± 0.09	0.25 ± 0.04^#^
P-Gsk-3β	0.34 ± 0.05	0.46 ± 0.05^#^	0.63 ± 0.05^*^
Gsk-3β	0.74 ± 0.05	0.85 ± 0.04^#^	0.93 ± 0.04^*^

*P < 0.01 vs. 0 mg/mL; ^#^P < 0.05 vs. 0 mg/mL.

### SBS inhibited the upregulation of Wnt5a and β-catenin induced by TGF-β1

**Figure 9:**
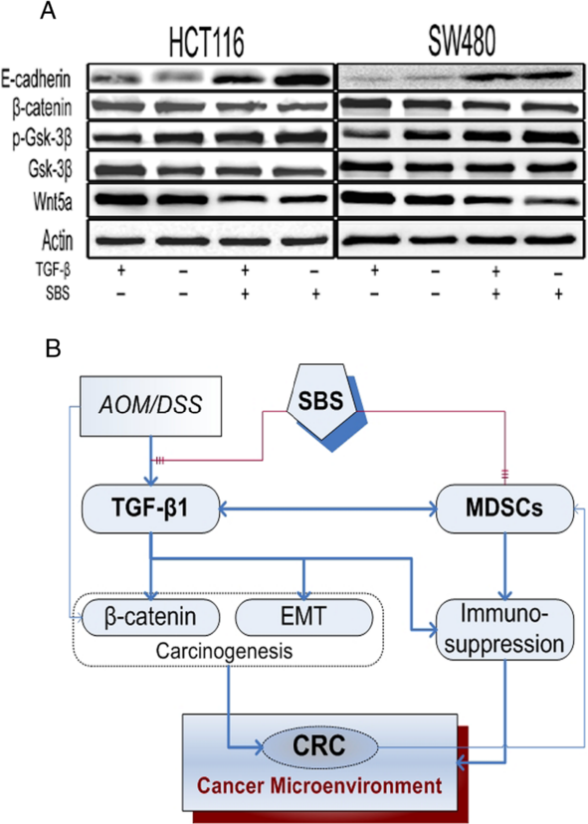
SBS inhibited the upregulation of Wnt5a and β-catenin induced by TGF-β1. **(A)** Western blot analysis of Wnt pathway molecules. 10 ng/mL TGF-β1 induced the expression of β-catenin and Wnt5a and dephosphotylated GSK-3β at ser 9. SBS reduced the expression of β-catenin and Wnt5a and increased the phosphorylation of GSK-3β in both HCT116 and SW480 cells. Semiquantitive analysis is showed in Table 6. **(B)** A diagram of effects of SBS on caCRC.

To further elucidate the mechanism of SBS in TGF-β1 mediated Wnt pathway, the expression of β-catenin and Wnt5a and phoshorylation of GSK-3β was examined. TGF-β1 induced the expression of β-catenin and Wnt5a and dephosphotylated GSK-3β at ser 9. SBS reduced the expression of β-catenin and Wnt5a and increased the phosphorylation of GSK-3β in both HCT116 and SW480 cells. It also reversed the downregulation of E-cad in cells treated with TGF-β1 (Figure 9, Table 6).

**Table 6:** **Semiquantitive analysis of Western blot on SW480 and HCT116**

	**T (n = 3)**	**C (n = 3)**	**T + S (n = 3)**	**S (n = 3)**
E-cadherin-H	0.16 ± 0.07	0.12 ± 0.04	0.58 ± 0.13^*^	1.54 ± 0.17
β-catenin-H	1.57 ± 0.05	1.36 ± 0.10	1.03 ± 0.10^*^	0.92 ± 0.08
p-Gsk-3β-H	0.84 ± 0.07	1.46 ± 0.12	1.24 ± 0.09^*^	1.65 ± 0.09
Gsk-3β-H	1.64 ± 0.08	1.48 ± 0.06	1.50 ± 0.03^#^	1.47 ± 0.06
Wnt5a-H	1.61 ± 0.08	1.21 ± 0.04	0.90 ± 0.13^*^	0.52 ± 0.10
E-cadherin-S	0.11 ± 0.03	0.16 ± 0.05	0.71 ± 0.16^*^	1.25 ± 0.17
β-catenin-S	1.63 ± 0.09	1.48 ± 0.04	1.25 ± 0.09^*^	1.15 ± 0.06
p-Gsk-3β-S	0.57 ± 0.16	1.06 ± 0.14	1.38 ± 0.07^*^	1.74 ± 0.11
Gsk-3β-S	1.57 ± 0.05	1.55 ± 0.04	1.53 ± 0.05	1.54 ± 0.08
Wnt5a-S	1.86 ± 0.13	1.59 ± 0.06	1.26 ± 0.09^*^	1.03 ± 0.09

T: 10 ng/mL TGF-β1; C: Control; T + S: 10 ng/mL TGF-β1 and 12 mg/mL SBS.

S: 12 mg/mL SBS; *P < 0.01 vs. T; ^#^P < 0.05 vs.T.

## Discussion

Azoxymethane (AOM) is a procarcinogen causes formation of O^6^-methylguanine upon metallic activation [18]. Repeated DSS administration causes chronic inflammation which mimics IBD. Three cycles of DSS in combination with a single injection of AOM resulted in 100% incidence of colonic neoplasms in mice in our research. The neoplasms occured mainly in the middle to distal colon which was the corresponding segment of DSS induced colitis [18]. Therefore, inflammation provides a suitable ground for the formation of neoplasms in the colon. Upon DSS administration, mass death in model group was delayed and alleviated by SBS treatment.

The AOM/DSS induced neoplasm resembles human caCRC in several aspects of its molecular pathogenesis, specifically in both increased PCNA and β-catenin expression and nuclear translocation of the latter. Wild-type p53 is a rapidly degraded protein with a short half-life and a low cellular level [19]. The accumulation of p53 in model group and translocation to nuclear are also features of caCRC for early loss of function of p53 [10]. SBS not only increased the survival rate of mice, reduced the incidence and multiplicity of colonic neoplasms, but also downregulated the expression of caCRC markers, such as PCNA, β-catenin and p53, suggested that SBS was highly efficacious in ameliorating caCRC [16].

TGF-β1 was upregulated in our caCRC mice. It induces a dose dependent increasing of β-Catenin protein levels and a up to 5 fold transcriptional activity of β-Catenin responsive promoters [20]. AOM is known to induce mutations in exon 3 of Ctnnb, which causes constitutive activation of the Wnt pathway by stabilizing β-catenin [18]. So, various mutations around the GSK-3β phosphorylation sites on β-catenin combined with the upregulation of TGF-β might be causative to β-catenin accumulation in our study [18,20]. The phosphorylation at Ser9 of GSK-3β might be result of TGF-β activation. Our result showed that β-catenin negatively correlated with p-GSK-3β. The result is consistent with the observation that increased repression of GSK-3β after TGF-β treatment [20].

TGF-β is potent inducer of epithelial-mesenchymal transition (EMT) in cell culture, especially in cancer-associated EMT [21]. It is a key process for invasion and metastasis of solid tumors [22]. There was well-documented evidence supporting the notion that development of EMT in colorectal cancer leads to an aggressive phenotype that may promote metastatic spread and augment treatment resistance [23]. Consistent with the previous reports [24,25], E-cadherin level was downregulated, Snail, Vimentin and Fibronectin were upregulated in our results. SBS lowers the level of Wnt5a and reduces the markers of EMT.

Wnt5a is an effector of TGF-β in breast cancer [26]. TGF-β was found to upregulate Wnt5a expression through direct formation of Smad complex or indirect activation of NF-κB [27]. Wnt5a treatment activated Snail and initiated the transition events [28,29]. So, it promoted EMT with downregulation of E-cad and upregulation of Vimentin [30]. The upregulated Wnt5a by TGF-β could be suppressed by SBS, suggested that SBS might inhibit EMT process through downregulating TGF-β [31].

EMT is an important cause for immune escape, including CRC [32]. Tumor stroma components are engaged in active molecular crosstalk that has serious implications for immunological recognition of tumor in shaping the microenvironment. Among stromal cells infiltrating tumors, MDSCs represented one of the most important players mediating immunosuppression [33]. Tumor-derived factors support MDSCs generation, expansion and function. Consequently, MDSCs release TGF-β and IL-10 facilitating the formation of an immunosuppressive environment. SBS reduced the number of MDSCs and inhibited the generation of TGF-β1 in the late stage of our experiment. MDSCs may participate in the producing of TGF-β which inhibits cytotoxic lymphocyte functions [13]. Together, SBS might reduce the production of TGF-β by MDSCs to ameliorate mesenchymal transition induced by AOM/DSS .

Advanced age, immunosuppressive cytokines from chronic inflammation, tumor-derived immunosuppressive factors and surrendered immune cells, such as MDSCs constitute CRC microenvironment [34,35]. In TCM, the key pathogenesis of CRC immunosuppressive microenvironment is “Spleen-deficiency” [36]. SBS ameliorates immunosuppressive microenvironment through invigorating Spleen and replenishing Qi, suggesting that the ancient TCM theory “invigorating Spleen” is effective in preventing caCRC. Panax ginseng Ginsenoside and polysaccharide prevented breast cancer metastasis and decreased gastric cancer cell migration and invasion through reducing the expression of EMT markers [37,38]. Korean red ginseng enhanced T cell function by inhibiting the immunosuppressive activity of MDSCs [39]. These evidences further support the efficacy of “invigorating Spleen” herbs in the prevention of cancers through regulating EMT and MDSCs.

## Conclusion

To conclude, AOM/DSS induce caCRC through upregulation of TGF-β1, which in turn activates Wnt5a and subsequent EMT. caCRC recruits MDSCs to generate an immunosuppressive microenvironment through TGF-β1. SBS decreases TGF-β1 mediated EMT and β-catenin activation to alleviate carcinogenesis. It also decreases MDSCs infiltration to ameliorate the immunosuppressive tumor microenvironment [40]. Further studies will be conducted to investigate how SBS regulates MDSCs and its underlying mechanism in preventing CRC growth and metastasis.

## Additional files

Additional file 1:Figure S1. Similarity analyses of chromatographic SBS samples. The Similarity Evaluation System for Chromatographic Fingerprint of TCM (2004 A edition) was used to evaluate the similarities of the 7 batches of SBS. After peak-picking, template-matching process, the peaks in the spectra were matched automatically. **Table S1.** Comparability result of reproducibility of SBS samples. The reference template was set finally for spectra peak difference and entire similarity evaluation. The similarities of repeatability were from 0.814 to 0.987. The results showed that the preparation process of SBS was reasonable. **Figure S2.** The four matched references in SBS sample. Determination of atractylenolide-1, atractylenolide −2, gensenoside Rb1 and Rc in SBS sample: as the four active compounds in *Atractylodes macrocephala* and *Panax ginseng* respectively, the determination of the four compounds by the same HPLC eluted system could cotroll the quality of SBS. Additional file 2:
**SBS (3.64 g/kg) was administered by gavage in SBS-L group, SBS (7.28 g/kg) in SBS-M group, and SBS (14.56 g/kg) in SBS-H group.** (A) Microscopic view of neoplasma in mice. SBS reduced the size of neoplasma in a dose-dependent manner. (B) Dose-depedent effects of SBS on MDSCs and serum TGF-β1.SBS decreased the number of MDSCs and reduced the content of TGF-β1 in serum in a dose-dependent manner. ^*^ *P* < 0.01 vs. model, ^#^ *P* < 0.05 vs. model; ^△^ *P* < 0.01 vs. control, ^▲^ *P* < 0.0 5 vs. control.

## Abbreviations

SBS: Shenling Baizhu San
CRC: Colorectal cancer
AOM: Azoxymethane
DSS: Dextran sodium sulfate
caCRC: Colitis associated CRC
TGF-β1: Transforming growth factor β1
ELISA: Enzyme-linked immunosorbent assay
EMT: Epithelial mesenchymal transition
N-cad: N-cadherin
E-cad: E-cadherin
MDSCs: Myeloid-derived suppressive cells
TCM: Traditional Chinese medicine
Dvl2: Dishevelled-2
GSK-3β: Glycogen synthase kinase 3β
