# Supplementary material for: Shenling Baizhu San supresses colitis associated colorectal cancer through inhibition of epithelial-mesenchymal transition and myeloid-derived suppressor infiltration
Source: BMC Complement Altern Med. 2015 Apr 22;15:126. doi: 10.1186/s12906-015-0649-9 (PMC4428101; doi:10.1186/s12906-015-0649-9)
Supplement: Additional file 1:Figure S1. — Similarity analyses of chromatographic SBS samples. The Similarity Evaluation System for Chromatographic Fingerprint of TCM (2004 A edition) was used to evaluate the similarities of the 7 batches of SBS. After peak-picking, template-matching process, the peaks in the spectra were matched automatically. Table S1. Comparability result of reproducibility of SBS samples. The reference template was set finally for spectra peak difference and entire similarity evaluation. The similarities of repeatability were from 0.814 to 0.987. The results showed that the preparation process of SBS was reasonable. Figure S2. The four matched references in SBS sample. Determination of atractylenolide-1, atractylenolide −2, gensenoside Rb1 and Rc in SBS sample: as the four active compounds in Atractylodes macrocephala and Panax ginseng respectively, the determination of the four compounds by the same HPLC eluted system could cotroll the quality of SBS. [file 12906_2015_649_MOESM1_ESM.doc]

Compositional analysis of SBS by HPLC

Instruments: High performance liquid chromatography (HPLC) analysis of SBS was performed on Shimadzu HPLC system (Shimadzu Co., Kyoto, Japan) using a C_18_ column Shimadzu VP ODS (250×4.6 mm; particle size 5 μm, Japan).

Method: The mobile phases comprised eluent A (0.5 ‰ aqueous acetic acid) and eluent B (acetonitrile). The gradient flow was as follows: 0.00–90.00 min, 10%–70% B; 90.01–105.00 min, 95% B; 105.01–110.00 min, 50% B; 110.01–120.00 min, 10% B. The analysis was performed at a flow rate of 1.0 mL/min with PDA detection at 215 nm. The injection volume was 25 μL.

The reproducibility of SBS samples: The Similarity Evaluation System for Chromatographic Fingerprint of TCM (2004 A edition) was used to evaluate the similarities of the 7 batches of SBS. After peak-picking, template-matching process, the peaks in the spectra were matched automatically (Fig. S1). The reference template was set finally for spectra peak difference and entire similarity evaluation. The similarities of repeatability were from 0.814 to 0.987 (Table S1)．The results showed that the preparation process of SBS was reasonable.

Determination of atractylenolide-1, atractylenolide -2, gensenoside Rb1 and Rc in SBS sample: as the four active compounds in *Atractylodes macrocephala* and *Panax ginseng* respectively, the determination of the three compounds by the same HPLC eluted system could cotroll the quality of SBS. (Fig. S2).

Fig. S1 Similarity analyses of chromatographic SBS samples

Table S1. Comparability result of reproducibility of SBS samples

|  | Sample 1 | Sample 2 | Sample 3 | Sample 4 | Sample 5 | Sample 6 | Sample 7 | Reference Fingerprint |
| --- | --- | --- | --- | --- | --- | --- | --- | --- |
| Sample 1 | 1.000 | 0.848 | 0.847 | 0.848 | 0.962 | 0.959 | 0.961 | 0.957 |
| Sample 2 | 0.848 | 1.000 | 0.987 | 0.985 | 0.841 | 0.828 | 0.820 | 0.951 |
| Sample 3 | 0.847 | 0.987 | 1.000 | 0.978 | 0.863 | 0.838 | 0.832 | 0.956 |
| Sample 4 | 0.848 | 0.985 | 0.978 | 1.000 | 0.842 | 0.827 | 0.814 | 0.948 |
| Sample 5 | 0.962 | 0.841 | 0.863 | 0.842 | 1.000 | 0.967 | 0.963 | 0.960 |
| Sample 6 | 0.959 | 0.828 | 0.838 | 0.827 | 0.967 | 1.000 | 0.993 | 0.956 |
| Sample 7 | 0.961 | 0.820 | 0.832 | 0.814 | 0.963 | 0.993 | 1.000 | 0.951 |
| Reference Fingerprint | 0.957 | 0.951 | 0.956 | 0.948 | 0.960 | 0.956 | 0.951 | 1.000 |

Fig. S2 The four matched references in SBS sample
