# Supplementary figures and images for: Shenling Baizhu San supresses colitis associated colorectal cancer through inhibition of epithelial-mesenchymal transition and myeloid-derived suppressor infiltration
Source: BMC Complement Altern Med. 2015 Apr 22;15:126. doi: 10.1186/s12906-015-0649-9 (PMC4428101; doi:10.1186/s12906-015-0649-9)

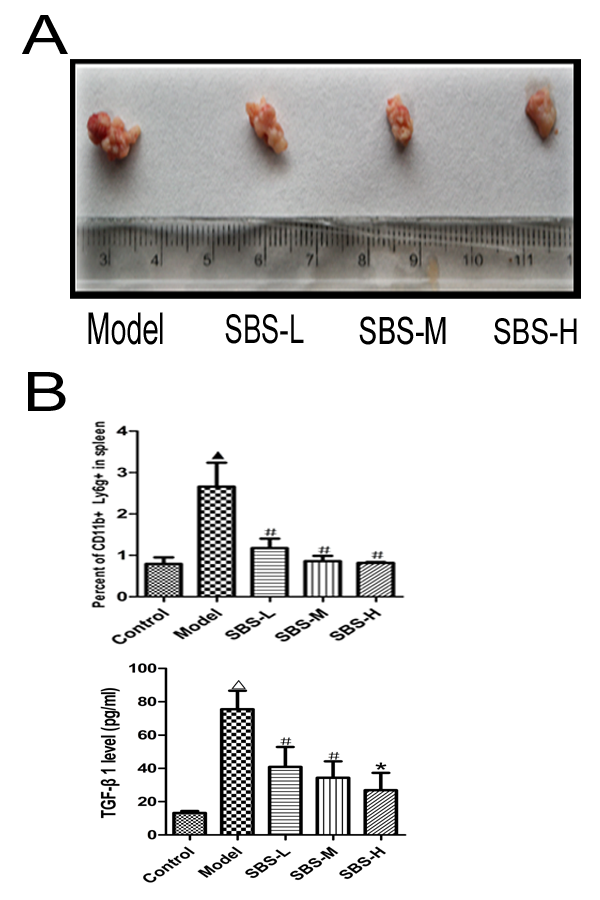

Supplement: Additional file 2: — SBS (3.64 g/kg) was administered by gavage in SBS-L group, SBS (7.28 g/kg) in SBS-M group, and SBS (14.56 g/kg) in SBS-H group. (A) Microscopic view of neoplasma in mice. SBS reduced the size of neoplasma in a dose-dependent manner. (B) Dose-depedent effects of SBS on MDSCs and serum TGF-β1.SBS decreased the number of MDSCs and reduced the content of TGF-β1 in serum in a dose-dependent manner. * P < 0.01 vs. model, # P < 0.05 vs. model; △ P < 0.01 vs. control, ▲ P < 0.0 5 vs. control. [file 12906_2015_649_MOESM2_ESM.tiff]
